# Supplementary material for: Impact of housing allowance programme on the physical and mental health of households in South Korea
Source: PLoS One. 2026 Feb 23;21(2):e0334490. doi: 10.1371/journal.pone.0334490 (PMC12928566; doi:10.1371/journal.pone.0334490)
Supplement: Attachment — Submitted filename: Response to Reviewers.docx [file pone.0334490.s002.docx]

**[PLOS ONE] Manuscript ID: PONE-D-25-23017**

**“Impact of housing allowance programme on the physical and mental health of households in South Korea”**

***Author's message****: Thank you for your valuable and constructive feedback, which has greatly enriched our study. We have carefully considered all comments and have substantially revised the manuscript in response. In particular, following the reviewers' suggestions, we have implemented a major revision of our analytical methodology to ensure a more rigorous causal analysis.*

*In the revised manuscript, we have highlighted all major additions and new content in yellow for clarity. Please note that due to the fundamental changes in our methodology, there have been corresponding revisions throughout the manuscript—from the introduction and methods to the results and discussion—that may extend beyond the highlighted sections.*

**Response to Editor’s Comments:**

**Editor’s Comments #1**

*Please ensure that your manuscript meets PLOS ONE's style requirements, including those for file naming.*

1) Thank you. We have carefully reviewed the PLOS ONE style guidelines and have revised the entire manuscript and its associated files to ensure full compliance with the formatting and file naming conventions.

**Editor’s Comments #2**

*Your study's underlying data set... does not qualify as an acceptable data repository... please upload the minimal data set necessary to replicate your study's findings to a stable, public repository...*

2) Thank you for this guidance. We have uploaded the minimal dataset necessary to replicate our findings to **Figshare**. The dataset is now publicly available and can be accessed via the following DOI: **[10.6084/m9.figshare.29938802]**.

**Editor’s Comments #3**

*PLOS requires an ORCID iD for the corresponding author... Please ensure that you have an ORCID iD and that it is validated in Editorial Manager.*

3) We confirm that the corresponding author has a validated ORCID iD, which has been linked and verified within the Editorial Manager system.

**Response to Reviewer 1’s Comments:**

**Reviewer 1’s Comments #1**

*More detail should be provided up front about what the housing allowance program actually does. For readers outside South Korea, there may not be familiarity with this type of program. Although the authors describe the program in the lit review section, more could be said in the introduction to motivate the study. It is not clear until page 5 whether this program is similar to the US housing voucher program or whether the subsidy is more open-ended. And even in this paragraph, the description of what the program actually provides is somewhat vague. Finally, even after getting to the end of this section, it was not entirely clear whether the housing allowance was provided as a cash transfer or in the form of a voucher. Finally, more detail should be provided on exactly how the expansion of the program worked in terms of the magnitude of what it provided.*

1) Thank you for this important suggestion to improve clarity, especially for our international readers. We agree that more upfront detail about the program was needed. To address this comprehensively, we have made significant revisions to both the Introduction and the Literature Review.

The Introduction now explicitly states that the Korean housing allowance is a direct cash transfer, providing a monthly subsidy to households. We have also added specific details about the magnitude of the 2015 program expansion, including the increase in the income eligibility threshold, to better motivate the study from the outset.

Furthermore, we have added a new paragraph in Section 2.1 that details the similarities and differences between the housing assistance programs in South Korea, the U.S., and the U.K. This new section clarifies that while Korea’s program is a tenant-based cash transfer, the U.S. system is more diverse, including both tenant-based vouchers and project-based assistance.

We believe these combined revisions now provide the necessary detail and international context that was previously missing. These changes can be found in the Introduction. (**Page 2, Line 21 ~ Page 3, Line 19; Page 4, Line 2 ~ Page 5, Line 8; Page 5, Line 19 ~ Page 6 Line 5**)

**Reviewer 1’s Comments #2**

*There should be a more thorough justification of the inclusion of some characteristics in the model. The DID model should difference out time-invariant differences between the treatment and control groups, so there should not be a need to control for these. If they are included to enhance statistical precision, this should be made clear. As for time-varying covariates that differ between treatment and control, more attention should be paid to why we would expect these not to be on the pathway from the policy to the outcome. Are some of the covariates included (e.g. smoking status) time varying? If so, this could create serious bias if treatment affects these variables.*

2) Thank you for this important methodological point. We have revised the manuscript to clarify the role of all covariates. As described in the revised **Section 3.2 (Variable setting)**, a rich set of pre-reform characteristics were used in the propensity score matching (PSM) model to construct a balanced control group and minimize selection bias. Subsequently, time-varying covariates were included in our final two-way fixed effects (TWFE) model primarily to improve the statistical precision of our event study estimates, a rationale now clarified in the text. (**Page 12, Lines 1 ~ 11**)

**Reviewer 1’s Comments #3**

*It is not clear to me who the control group is. Is it just households not receiving the housing allowance? How is this determined? Why would we expect trends in this group to be similar to those of the treatment group?*

3) We agree that our initial control group was not well-defined. This was a critical issue that we have now addressed with a fundamental revision of our research design. As detailed in **Section 3.1 and 4.1**, we now employ propensity score matching (PSM) to construct a highly comparable control group of non-recipients. The success of this matching is demonstrated in Table 1 and Figure 1, which show that the two groups are well-balanced across a wide range of observable characteristics after matching. This robust process ensures that the control group is a valid counterfactual. (**Page 10, Lines 14 ~ 20**)

**Reviewer 1’s Comments #4**

*Similarly, is receiving the housing allowance a time-varying variable? If so, are some respondents receiving the allowance after 2015 but not before?*

4) Yes, that is correct. We have clarified this in **Section 3.1**. The underlying data is a panel where a household's receipt status can change year-to-year. For our analysis, we define the "treatment group" as any household that has ever received the allowance during the study period, which allows us to observe households transitioning into the program after the 2015 reform. (**Page 10, Lines 10 ~ 13**)

**Reviewer 1’s Comments #5**

*At the end of the first paragraph of the Data Analysis and Methodology section, there is a sentence that mentions that data deemed outliers were excluded, but there is no more detail about how this was determined. Outliers on which characteristics? How many were outliers? Just because an individual is an outlier, does not mean they should be excluded. Much more detail is needed.*

5) Thank you for pointing out this lack of transparency. We have replaced the vague statement with a specific and reproducible rule. The revised **Section 3.1** now states that we handled outliers by "winsorizing continuous variables at the 0.5th and 99.5th percentiles." (**Page 10, Lines 8 ~ 10**)

**Reviewer 1’s Comments #6**

*DID assumes that—in the absence of treatment—trends for the treatment and control group would be identical. The parallel trends assumption cannot be tested, but must be justified based on theory and supporting empirical results. Authors often show event study results to confirm that treatment effects indeed only show up after the treatment. Something like this would go a long way to assuage my concerns that the models are simply picking up unrelated background trends.*

6) We completely agree with this crucial point. We have now made an **event study analysis** a central part of our methodology. First, in **Section 4.1**, we present **Figure 2**, which visually shows that the raw trends were remarkably parallel before the reform. Second, in **Section 4.2**, we present the formal results of our TWFE event study model. **Figure 3** and **Table 2** clearly show that the coefficients for all pre-treatment years are statistically insignificant, providing strong empirical evidence that the parallel trends assumption is satisfied. (**Pages 15 ~ 20**)

**Reviewer 1’s Comments #7**

*In Table 6, the coefficient for the DID variable is 0.08 and the standard error is 0.02, but it is not listed as significant.*

7) Thank you for this careful observation. The original analysis and tables (including the former Table 6) have been completely replaced by the new, more rigorous TWFE event study analysis presented in the revised Table 2. We have meticulously reviewed the new results in Table 2 to ensure all coefficients and significance levels are reported accurately.

**Reviewer 1’s Comments #8**

*No attempt is made in the results section to interpret the effect size. All statements are directional, but it is not clear if the effect is meaningfully large or reasonably sized.*

8) This is an excellent suggestion. The revised **Section 4.2** and **Section 5 (Discussion)** now include a practical interpretation of the effect sizes. We particularly discuss the nuance that the effects are not immediate but emerge and accumulate over several years, and we frame these delayed benefits as a "long-term investment in public health."

**Reviewer 1’s Comments #9**

*DID models should also include dummy variables for individual cases. Although those variables may be in the model, it is not clear from Tables 5 and 6 whether this is the case.*

9) Thank you for this important clarification request. Our revised analysis now explicitly uses a two-way fixed effects (TWFE) model, which includes individual (household) fixed effects. This directly addresses the concern about controlling for time-invariant unobserved characteristics. We have clearly stated this in the **Methodology (Section 3.1)**, and the main results table (**Table 2**) is now explicitly labeled as a TWFE model.

**Response to Reviewer 2’s Comments:**

**Reviewer 2’s Comments #1**

*Consider citing "Bhat, A. C., Fenelon, A., & Almeida, D. M. (2025). Housing insecurity pathways to physiological and epigenetic manifestations of health among aging adults: a conceptual model. Frontiers in Public Health, 13, 1485371." to provide a conceptual model basis for how housing assistance programs can ameliorate the adverse health impacts of housing insecurity.*

1) Thank you for this valuable literature suggestion. We have incorporated the recommended paper by Bhat et al. into our Literature Review section. It provides an excellent conceptual framework for understanding the pathways through which housing security can impact health, strengthening the theoretical foundation of our study. (**Page 6, Line 15 ~ Page 7, Line 4**)

**Reviewer 2’s Comments #2**

*Please add more literature to support the disproportionate impacts on women/ethnic minorities/older adults. What are the factors that contribute to vulnerabilities for these groups? Also explain how this ties into the analyses you are conducting in the context of Korea.*

2) Thank you for this constructive feedback. Following your suggestion, we have substantially expanded the literature review to better support the disproportionate impacts of housing cost burdens. The revised section now incorporates a broader range of international literature detailing the specific vulnerabilities of women, older adults, and other at-risk groups, and explains the factors contributing to these challenges. Crucially, the revised paragraph now concludes by explicitly connecting this international evidence to our study’s specific context and analytical variables (gender and age) in Korea. **(Page 7, Line 21 ~ Page 8, Line 21)**

**Reviewer 2’s Comments #3**

*Both discussion and review of literature sections are a bit thin. There are several more studies than what you cited that discuss how governmental assistance programs ameliorate housing insecurity and related health effects (physical and mental health). Look into more of the work by Andrew Fenelon and others who have published extensively in this area and incorporate into these sections.*

3) We thank the reviewer for this constructive feedback. We have substantially revised and expanded both the Literature Review and Discussion sections. We have integrated several key studies, including those by Andrew Fenelon, to better situate our findings within the broader international literature on housing assistance and health. This has allowed for a much richer and more nuanced discussion of our results. (**Page 8, Line 22 ~ Page 9, Line 10; Page 21, Lines 2 ~ 13**)

**Reviewer 2’s Comments #4**

*Go more into depth regarding the similarities and differences in housing assistance programs in countries such as the US/UK and Korea*

4) Following the reviewer's suggestion, we have added a more detailed comparison in the Discussion section. We now contrast the direct cash-transfer nature of the Korean program with the often voucher-based systems in the US and UK, and we discuss how these structural differences might lead to different outcomes for recipients. (**Page 4, Line 20 ~ Page 5, Line 8**)

**Reviewer 2’s Comments #5**

*In the US access to housing assistance programs also increases access to health insurance (Simon, A. E., Fenelon, A., Helms, V., Lloyd, P. C., & Rossen, L. M. (2017). HUD housing assistance associated with lower uninsurance rates and unmet medical need. Health Affairs, 36(6), 1016-1023.). Would this be expected to translate similarly in the context of Korea?*

5) Thank you for this very insightful question which highlights a critical contextual difference. The reviewer is correct to point out that in the U.S., increased access to health insurance is a key pathway through which housing assistance improves health (Simon et al., 2017).

However, this mechanism is not expected to translate similarly to the context of South Korea due to a fundamental difference in healthcare systems. South Korea operates a universal National Health Insurance (NHI) system that covers all citizens. Therefore, unlike in the U.S., access to basic health insurance is not a significant variable that changes upon receipt of the housing allowance, as most, if not all, of our study participants would have been insured regardless of their housing status.

This distinction makes our findings particularly valuable, as they can isolate other crucial pathways free from the confounding effect of gaining insurance access. We believe that in the Korean context, the observed health improvements are more likely driven by the direct reduction of financial stress, improved housing stability, and an increase in mental "bandwidth" that allows households to better prioritize their health management.

**Reviewer 1’s Comments #6**

*Consider whether only examining household heads' health is a limiting factor, rather than examining health of multiple members of the household. Write about this in limitations.*

6) We agree this is an important limitation. We have added a statement to the Limitations section acknowledging that our focus on the household head means our findings may not be generalizable to other household members, such as children or spouses, whose health may be affected differently by the program. We suggest this as an avenue for future research. (**Page 21, Line 24 ~ Page 22, Line 11**).
