# Supplementary material for: Impact of housing allowance programme on the physical and mental health of households in South Korea
Source: PLoS One. 2026 Feb 23;21(2):e0334490. doi: 10.1371/journal.pone.0334490 (PMC12928566; doi:10.1371/journal.pone.0334490)
Supplement: Attachment — Submitted filename: Response_to_Reviewers_auresp_2.docx [file pone.0334490.s003.docx]

**[PLOS ONE] Manuscript ID: PONE-D-25-23017**

**“Impact of housing allowance programme on the physical and mental health of households in South Korea”**

***Author's message****: We thank the editor and reviewers again for their time and valuable contributions, which have helped us improve the quality of our manuscript. We hope the revised version is now suitable for publication in PLOS One. In the revised manuscript, we have highlighted all major additions and new content in yellow for clarity.*

**Response to Reviewer 3’s Comments:**

**Reviewer 3’s Comments #1**

*Page 12 Line 2-4: The sentence could be improved e.g. A TWFE event study replaces a single pre-post treatment indicator with a series of year-by-year interaction terms. This approach allows researchers to formally test the key parallel trends assumption and to trace the dynamic, year-over-year effects of the policy following the 2015 reform (Clarke 5 & Tapia-Schythe, 2021; Sun & Abraham, 2021).*

1) Thank you for this excellent suggestion. The proposed wording is indeed clearer and improves the flow of the methodology section. We have revised the text in Section 3.1 (Data and methodology) to incorporate your suggested sentences precisely as recommended. (**Page 11, Line 2 ~ 5**)

**Reviewer 3’s Comments #2**

*The statistical software used, including its publisher and version, along with the level of significant applied are to be stated.*

2) Thank you for pointing this out. We have added a statement at the end of Section 3.1 (Data and methodology) to clarify the software and significance levels used. The added sentence reads:

*"All statistical analyses were conducted using Python version 3.12. Statistical significance was defined at the p < 0.1, p < 0.05, and p < 0.01 levels, as indicated in the tables."* (**Page 11, Lines 16 ~ 17**)

**Reviewer 3’s Comments #3**

*Table 2: Adjusted R^2, RMSE, F-Statistic could be presented.*

3) We agree that these model fit statistics are important for assessing the models. We have updated the 'Model statistics' section of Table 2 to include the Adjusted R-squared (Within), RMSE, and F-Statistic for both the mental and physical health models. (**Page 19, Table 2**)

**Reviewer 3’s Comments #4**

*The assumptions fulfilment for the statistical test are to be mentioned.*

4) This is a crucial point. To explicitly address the assumptions of our analytical strategy, we have added a new, dedicated paragraph at the end of Section 3.1 (Data and methodology). This new paragraph outlines the two key assumptions—the conditional independence assumption for PSM and the parallel trends assumption for the TWFE model—and details how we validated them (via SMD balance checks and pre-treatment trend analysis, with results presented in Sections 4.1 and 4.2). (**Page 11, Lines 7 ~ 15**)

**Reviewer 3’s Comments #5**

*Table 1, 2: The N for the intervention group and control group are to be stated.*

5) We appreciate this suggestion for improving clarity. We have made the following two changes:

1. In Table 1, we have updated the column headers to include the number of households (N) for both 'Before Matching' (Control group (N=3184), Treated group (N=302)) and 'After Matching' (Control group (N=278), Treated group (N=278)).

2. In Table 2, we have added the 'Number of Observations (4,951)' and 'Number of Households (556)' to the 'Model statistics' section to clarify the panel data structure used in the regression analysis.

(**Page 14, Table 1; Page 19, Table 2**)

**Reviewer 3’s Comments #6**

*All references in the text and the reference list are to follow journal’s format.*

6) We have thoroughly reformatted the entire manuscript to comply with PLOS One's citation guidelines. All in-text citations have been converted to the required numeric, bracketed format (e.g., [1]), and the reference list has been re-ordered and re-formatted based on the order of appearance.
